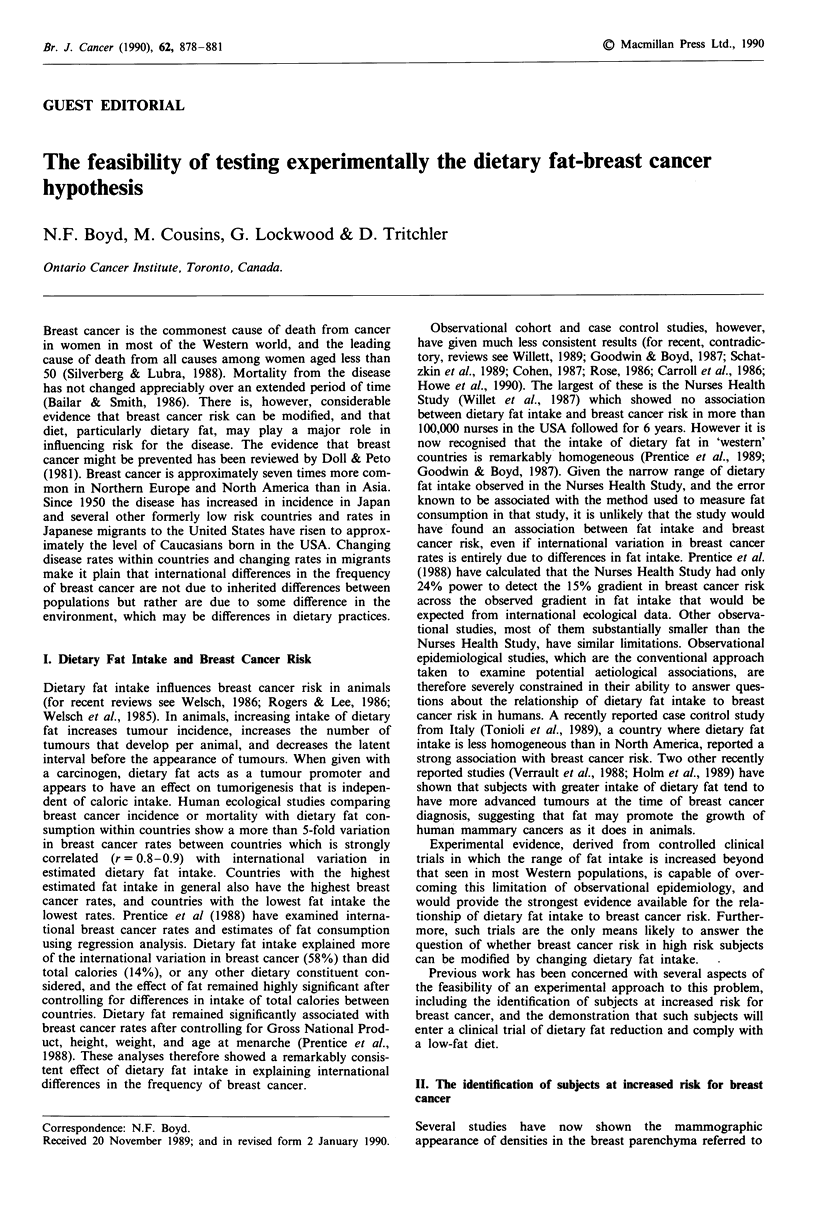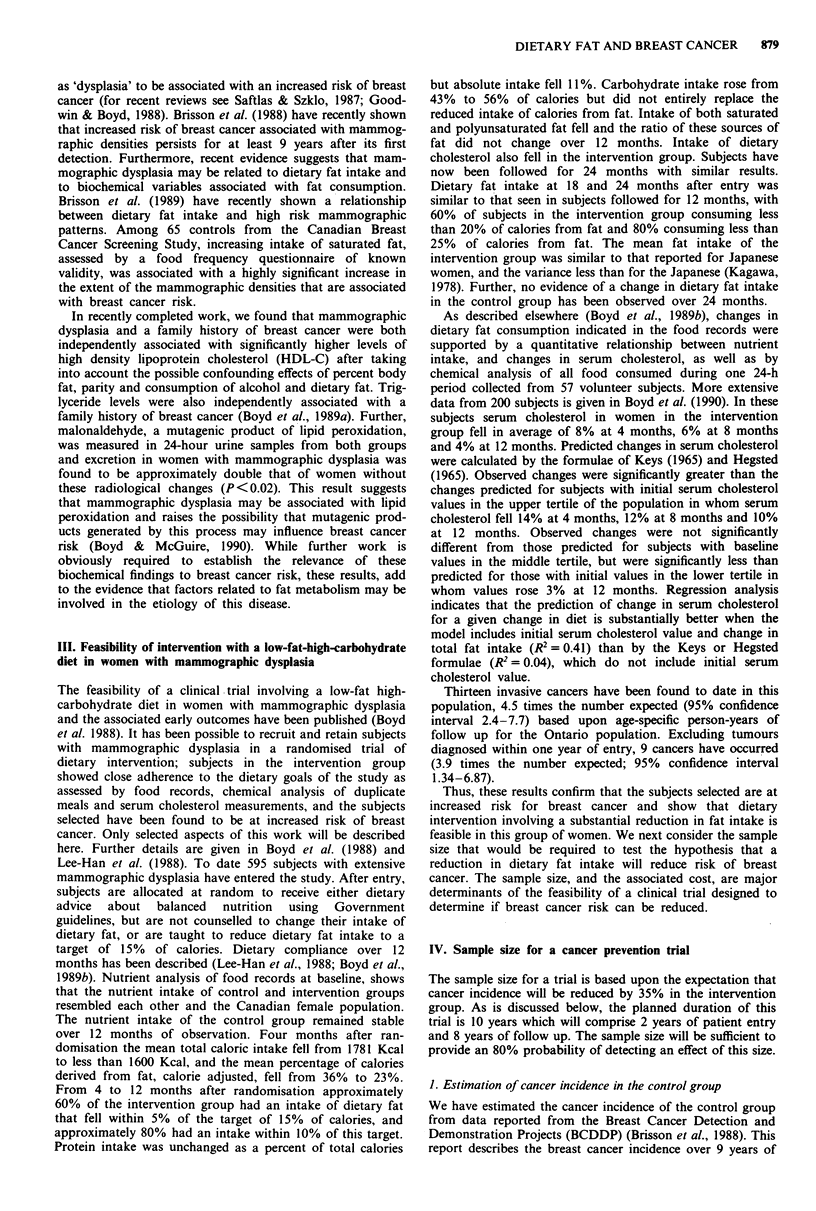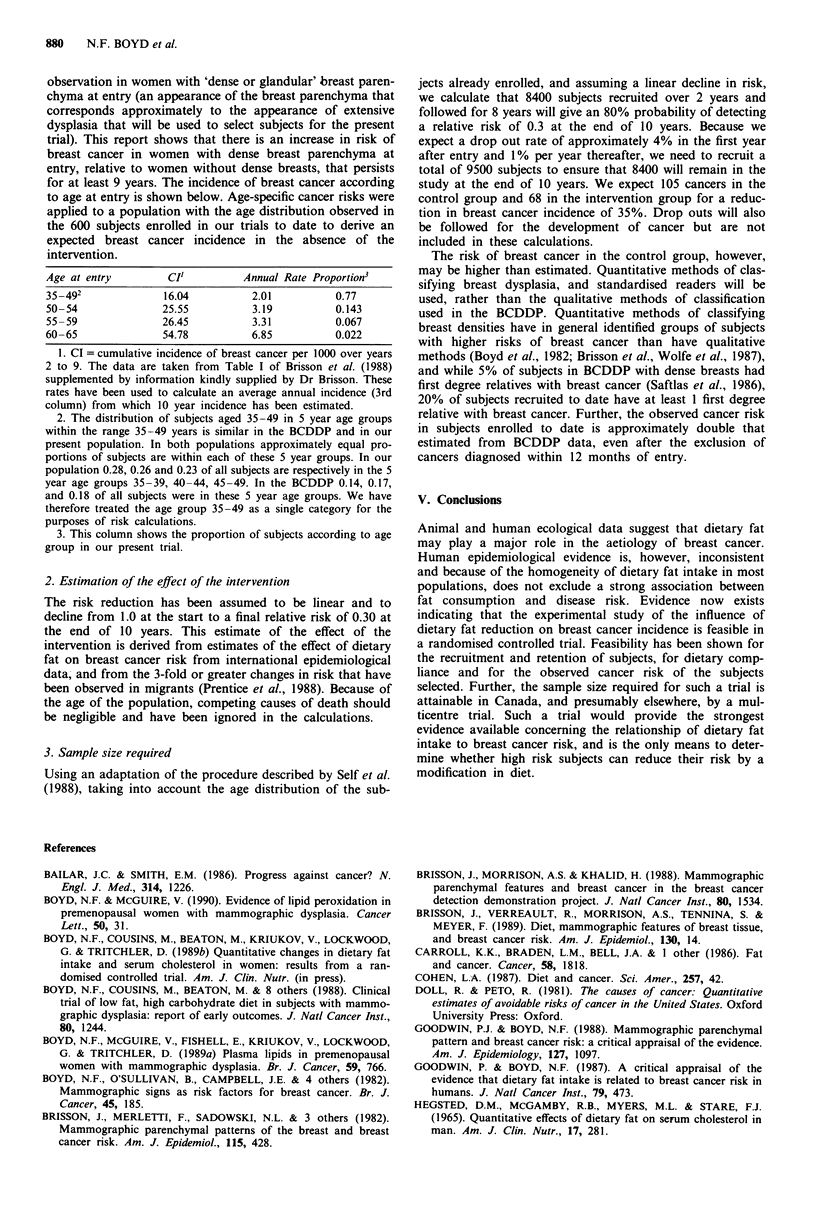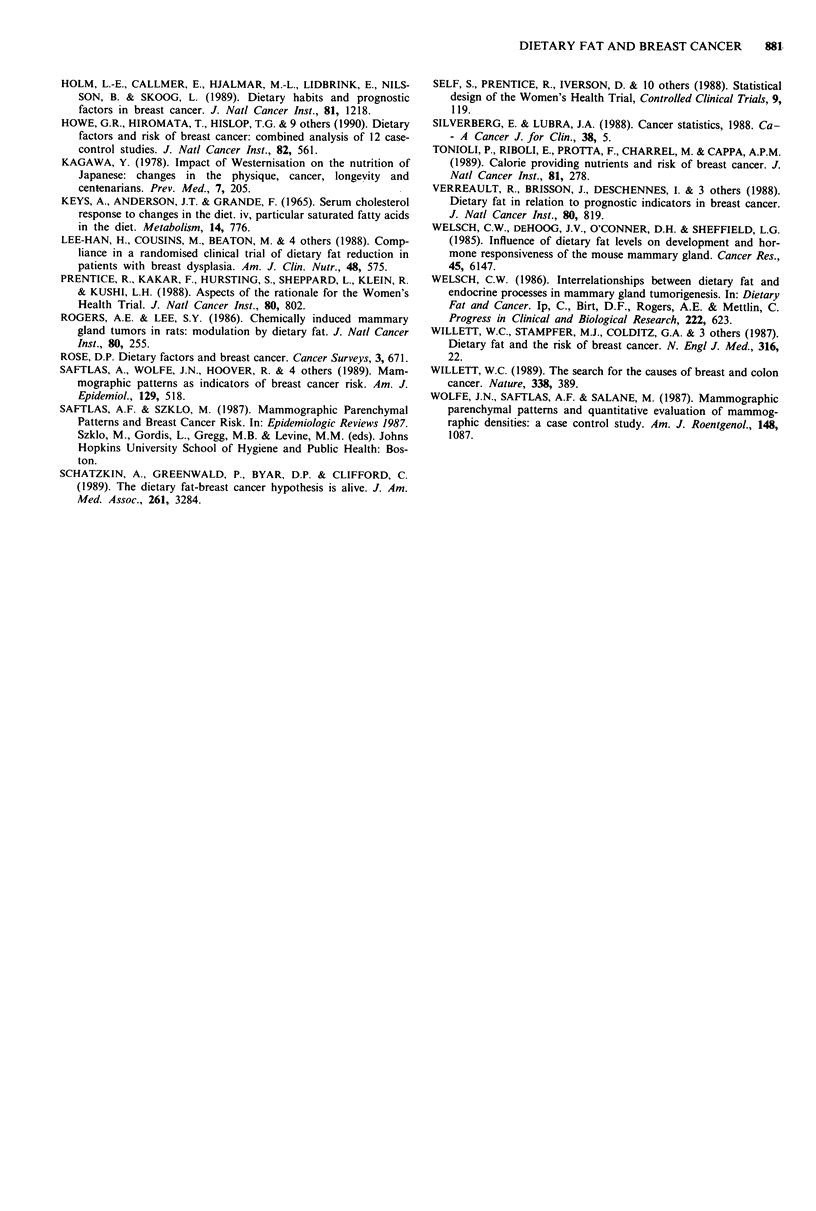# The feasibility of testing experimentally the dietary fat-breast cancer hypothesis.

**DOI:** 10.1038/bjc.1990.400

**Published:** 1990-12

**Authors:** N. F. Boyd, M. Cousins, G. Lockwood, D. Tritchler


Br. J. Cancer (1990), 62, 878–881 © Macmillan Press Ltd., 1990

GUEST EDITORIAL

### The feasibility of testing experimentally the dietary fat-breast cancer hypothesis

N.F. Boyd, M. Cousins, G. Lockwood & D. Tritchler

*Ontario Cancer Institute, Toronto, Canada.*

Breast cancer is the commonest cause of death from cancer in women in most of the Western world, and the leading cause of death from all causes among women aged less than 50 (Silverberg & Lubra, 1988). Mortality from the disease has not changed appreciably over an extended period of time (Bailar & Smith, 1986). There is, however, considerable evidence that breast cancer risk can be modified, and that diet, particularly dietary fat, may play a major role in influencing risk for the disease. The evidence that breast cancer might be prevented has been reviewed by Doll & Peto (1981). Breast cancer is approximately seven times more common in Northern Europe and North America than in Asia. Since 1950 the disease has increased in incidence in Japan and several other formerly low risk countries and rates in Japanese migrants to the United States have risen to approximately the level of Caucasians born in the USA. Changing disease rates within countries and changing rates in migrants make it plain that international differences in the frequency of breast cancer are not due to inherited differences between populations but rather are due to some difference in the environment, which may be differences in dietary practices.

### I. Dietary Fat Intake and Breast Cancer Risk

Dietary fat intake influences breast cancer risk in animals (for recent reviews see Welsch, 1986; Rogers & Lee, 1986; Welsch *et al.*, 1985). In animals, increasing intake of dietary fat increases tumour incidence, increases the number of tumours that develop per animal, and decreases the latent interval before the appearance of tumours. When given with a carcinogen, dietary fat acts as a tumour promoter and appears to have an effect on tumorigenesis that is independent of caloric intake. Human ecological studies comparing breast cancer incidence or mortality with dietary fat consumption within countries show a more than 5-fold variation in breast cancer rates between countries which is strongly correlated ($r = 0.8–0.9$) with international variation in estimated dietary fat intake. Countries with the highest estimated fat intake in general also have the highest breast cancer rates, and countries with the lowest fat intake the lowest rates. Prentice *et al* (1988) have examined international breast cancer rates and estimates of fat consumption using regression analysis. Dietary fat intake explained more of the international variation in breast cancer (58%) than did total calories (14%), or any other dietary constituent considered, and the effect of fat remained highly significant after controlling for differences in intake of total calories between countries. Dietary fat remained significantly associated with breast cancer rates after controlling for Gross National Product, height, weight, and age at menarche (Prentice *et al.*, 1988). These analyses therefore showed a remarkably consistent effect of dietary fat intake in explaining international differences in the frequency of breast cancer.

Correspondence: N.F. Boyd.
Received 20 November 1989; and in revised form 2 January 1990.

Observational cohort and case control studies, however, have given much less consistent results (for recent, contradictory, reviews see Willett, 1989; Goodwin & Boyd, 1987; Schatzkin *et al.*, 1989; Cohen, 1987; Rose, 1986; Carroll *et al.*, 1986; Howe *et al.*, 1990). The largest of these is the Nurses Health Study (Willet *et al.*, 1987) which showed no association between dietary fat intake and breast cancer risk in more than 100,000 nurses in the USA followed for 6 years. However it is now recognised that the intake of dietary fat in 'western' countries is remarkably homogeneous (Prentice *et al.*, 1989; Goodwin & Boyd, 1987). Given the narrow range of dietary fat intake observed in the Nurses Health Study, and the error known to be associated with the method used to measure fat consumption in that study, it is unlikely that the study would have found an association between fat intake and breast cancer risk, even if international variation in breast cancer rates is entirely due to differences in fat intake. Prentice *et al.* (1988) have calculated that the Nurses Health Study had only 24% power to detect the 15% gradient in breast cancer risk across the observed gradient in fat intake that would be expected from international ecological data. Other observational studies, most of them substantially smaller than the Nurses Health Study, have similar limitations. Observational epidemiological studies, which are the conventional approach taken to examine potential aetiological associations, are therefore severely constrained in their ability to answer questions about the relationship of dietary fat intake to breast cancer risk in humans. A recently reported case control study from Italy (Tonioli *et al.*, 1989), a country where dietary fat intake is less homogeneous than in North America, reported a strong association with breast cancer risk. Two other recently reported studies (Verrault *et al.*, 1988; Holm *et al.*, 1989) have shown that subjects with greater intake of dietary fat tend to have more advanced tumours at the time of breast cancer diagnosis, suggesting that fat may promote the growth of human mammary cancers as it does in animals.

Experimental evidence, derived from controlled clinical trials in which the range of fat intake is increased beyond that seen in most Western populations, is capable of overcoming this limitation of observational epidemiology, and would provide the strongest evidence available for the relationship of dietary fat intake to breast cancer risk. Furthermore, such trials are the only means likely to answer the question of whether breast cancer risk in high risk subjects can be modified by changing dietary fat intake.

Previous work has been concerned with several aspects of the feasibility of an experimental approach to this problem, including the identification of subjects at increased risk for breast cancer, and the demonstration that such subjects will enter a clinical trial of dietary fat reduction and comply with a low-fat diet.

### II. The identification of subjects at increased risk for breast cancer

Several studies have now shown the mammographic appearance of densities in the breast parenchyma referred to as 'dysplasia' to be associated with an increased risk of breast cancer (for recent reviews see Saftlas & Szklo, 1987; Goodwin & Boyd, 1988). Brisson *et al.* (1988) have recently shown that increased risk of breast cancer associated with mammographic densities persists for at least 9 years after its first detection. Furthermore, recent evidence suggests that mammographic dysplasia may be related to dietary fat intake and to biochemical variables associated with fat consumption. Brisson *et al.* (1989) have recently shown a relationship between dietary fat intake and high risk mammographic patterns. Among 65 controls from the Canadian Breast Cancer Screening Study, increasing intake of saturated fat, assessed by a food frequency questionnaire of known validity, was associated with a highly significant increase in the extent of the mammographic densities that are associated with breast cancer risk.

In recently completed work, we found that mammographic dysplasia and a family history of breast cancer were both independently associated with significantly higher levels of high density lipoprotein cholesterol (HDL-C) after taking into account the possible confounding effects of percent body fat, parity and consumption of alcohol and dietary fat. Triglyceride levels were also independently associated with a family history of breast cancer (Boyd *et al.*, 1989*a*). Further, malonaldehyde, a mutagenic product of lipid peroxidation, was measured in 24-hour urine samples from both groups and excretion in women with mammographic dysplasia was found to be approximately double that of women without these radiological changes ($P<0.02$). This result suggests that mammographic dysplasia may be associated with lipid peroxidation and raises the possibility that mutagenic products generated by this process may influence breast cancer risk (Boyd & McGuire, 1990). While further work is obviously required to establish the relevance of these biochemical findings to breast cancer risk, these results, add to the evidence that factors related to fat metabolism may be involved in the etiology of this disease.

### III. Feasibility of intervention with a low-fat-high-carbohydrate diet in women with mammographic dysplasia

The feasibility of a clinical trial involving a low-fat high-carbohydrate diet in women with mammographic dysplasia and the associated early outcomes have been published (Boyd *et al.* 1988). It has been possible to recruit and retain subjects with mammographic dysplasia in a randomised trial of dietary intervention; subjects in the intervention group showed close adherence to the dietary goals of the study as assessed by food records, chemical analysis of duplicate meals and serum cholesterol measurements, and the subjects selected have been found to be at increased risk of breast cancer. Only selected aspects of this work will be described here. Further details are given in Boyd *et al.* (1988) and Lee-Han *et al.* (1988). To date 595 subjects with extensive mammographic dysplasia have entered the study. After entry, subjects are allocated at random to receive either dietary advice about balanced nutrition using Government guidelines, but are not counselled to change their intake of dietary fat, or are taught to reduce dietary fat intake to a target of 15% of calories. Dietary compliance over 12 months has been described (Lee-Han *et al.*, 1988; Boyd *et al.*, 1989*b*). Nutrient analysis of food records at baseline, shows that the nutrient intake of control and intervention groups resembled each other and the Canadian female population. The nutrient intake of the control group remained stable over 12 months of observation. Four months after randomisation the mean total caloric intake fell from 1781 Kcal to less than 1600 Kcal, and the mean percentage of calories derived from fat, calorie adjusted, fell from 36% to 23%. From 4 to 12 months after randomisation approximately 60% of the intervention group had an intake of dietary fat that fell within 5% of the target of 15% of calories, and approximately 80% had an intake within 10% of this target. Protein intake was unchanged as a percent of total calories but absolute intake fell 11%. Carbohydrate intake rose from 43% to 56% of calories but did not entirely replace the reduced intake of calories from fat. Intake of both saturated and polyunsaturated fat fell and the ratio of these sources of fat did not change over 12 months. Intake of dietary cholesterol also fell in the intervention group. Subjects have now been followed for 24 months with similar results. Dietary fat intake at 18 and 24 months after entry was similar to that seen in subjects followed for 12 months, with 60% of subjects in the intervention group consuming less than 20% of calories from fat and 80% consuming less than 25% of calories from fat. The mean fat intake of the intervention group was similar to that reported for Japanese women, and the variance less than for the Japanese (Kagawa, 1978). Further, no evidence of a change in dietary fat intake in the control group has been observed over 24 months.

As described elsewhere (Boyd *et al.*, 1989*b*), changes in dietary fat consumption indicated in the food records were supported by a quantitative relationship between nutrient intake, and changes in serum cholesterol, as well as by chemical analysis of all food consumed during one 24-h period collected from 57 volunteer subjects. More extensive data from 200 subjects is given in Boyd *et al.* (1990). In these subjects serum cholesterol in women in the intervention group fell in average of 8% at 4 months, 6% at 8 months and 4% at 12 months. Predicted changes in serum cholesterol were calculated by the formulae of Keys (1965) and Hegsted (1965). Observed changes were significantly greater than the changes predicted for subjects with initial serum cholesterol values in the upper tertile of the population in whom serum cholesterol fell 14% at 4 months, 12% at 8 months and 10% at 12 months. Observed changes were not significantly different from those predicted for subjects with baseline values in the middle tertile, but were significantly less than predicted for those with initial values in the lower tertile in whom values rose 3% at 12 months. Regression analysis indicates that the prediction of change in serum cholesterol for a given change in diet is substantially better when the model includes initial serum cholesterol value and change in total fat intake ($R^2 = 0.41$) than by the Keys or Hegsted formulae ($R^2 = 0.04$), which do not include initial serum cholesterol value.

Thirteen invasive cancers have been found to date in this population, 4.5 times the number expected (95% confidence interval 2.4–7.7) based upon age-specific person-years of follow up for the Ontario population. Excluding tumours diagnosed within one year of entry, 9 cancers have occurred (3.9 times the number expected; 95% confidence interval 1.34–6.87).

Thus, these results confirm that the subjects selected are at increased risk for breast cancer and show that dietary intervention involving a substantial reduction in fat intake is feasible in this group of women. We next consider the sample size that would be required to test the hypothesis that a reduction in dietary fat intake will reduce risk of breast cancer. The sample size, and the associated cost, are major determinants of the feasibility of a clinical trial designed to determine if breast cancer risk can be reduced.

### IV. Sample size for a cancer prevention trial

The sample size for a trial is based upon the expectation that cancer incidence will be reduced by 35% in the intervention group. As is discussed below, the planned duration of this trial is 10 years which will comprise 2 years of patient entry and 8 years of follow up. The sample size will be sufficient to provide an 80% probability of detecting an effect of this size.

### *1. Estimation of cancer incidence in the control group*

We have estimated the cancer incidence of the control group from data reported from the Breast Cancer Detection and Demonstration Projects (BCDDP) (Brisson *et al.*, 1988). This report describes the breast cancer incidence over 9 years of observation in women with 'dense or glandular' breast parenchyma at entry (an appearance of the breast parenchyma that corresponds approximately to the appearance of extensive dysplasia that will be used to select subjects for the present trial). This report shows that there is an increase in risk of breast cancer in women with dense breast parenchyma at entry, relative to women without dense breasts, that persists for at least 9 years. The incidence of breast cancer according to age at entry is shown below. Age-specific cancer risks were applied to a population with the age distribution observed in the 600 subjects enrolled in our trials to date to derive an expected breast cancer incidence in the absence of the intervention.

| *Age at entry* | *CI*[1] | *Annual Rate* | *Proportion*[3] |
|---|---|---|---|
| 35–49[2] | 16.04 | 2.01 | 0.77 |
| 50–54 | 25.55 | 3.19 | 0.143 |
| 55–59 | 26.45 | 3.31 | 0.067 |
| 60–65 | 54.78 | 6.85 | 0.022 |

1. CI = cumulative incidence of breast cancer per 1000 over years 2 to 9. The data are taken from Table I of Brisson *et al.* (1988) supplemented by information kindly supplied by Dr Brisson. These rates have been used to calculate an average annual incidence (3rd column) from which 10 year incidence has been estimated.

2. The distribution of subjects aged 35–49 in 5 year age groups within the range 35–49 years is similar in the BCDDP and in our present population. In both populations approximately equal proportions of subjects are within each of these 5 year groups. In our population 0.28, 0.26 and 0.23 of all subjects are respectively in the 5 year age groups 35–39, 40–44, 45–49. In the BCDDP 0.14, 0.17, and 0.18 of all subjects were in these 5 year age groups. We have therefore treated the age group 35–49 as a single category for the purposes of risk calculations.

3. This column shows the proportion of subjects according to age group in our present trial.

### 2. *Estimation of the effect of the intervention*

The risk reduction has been assumed to be linear and to decline from 1.0 at the start to a final relative risk of 0.30 at the end of 10 years. This estimate of the effect of the intervention is derived from estimates of the effect of dietary fat on breast cancer risk from international epidemiological data, and from the 3-fold or greater changes in risk that have been observed in migrants (Prentice *et al.*, 1988). Because of the age of the population, competing causes of death should be negligible and have been ignored in the calculations.

### 3. *Sample size required*

Using an adaptation of the procedure described by Self *et al.* (1988), taking into account the age distribution of the subjects already enrolled, and assuming a linear decline in risk, we calculate that 8400 subjects recruited over 2 years and followed for 8 years will give an 80% probability of detecting a relative risk of 0.3 at the end of 10 years. Because we expect a drop out rate of approximately 4% in the first year after entry and 1% per year thereafter, we need to recruit a total of 9500 subjects to ensure that 8400 will remain in the study at the end of 10 years. We expect 105 cancers in the control group and 68 in the intervention group for a reduction in breast cancer incidence of 35%. Drop outs will also be followed for the development of cancer but are not included in these calculations.

The risk of breast cancer in the control group, however, may be higher than estimated. Quantitative methods of classifying breast dysplasia, and standardised readers will be used, rather than the qualitative methods of classification used in the BCDDP. Quantitative methods of classifying breast densities have in general identified groups of subjects with higher risks of breast cancer than have qualitative methods (Boyd *et al.*, 1982; Brisson *et al.*, Wolfe *et al.*, 1987), and while 5% of subjects in BCDDP with dense breasts had first degree relatives with breast cancer (Saftlas *et al.*, 1986), 20% of subjects recruited to date have at least 1 first degree relative with breast cancer. Further, the observed cancer risk in subjects enrolled to date is approximately double that estimated from BCDDP data, even after the exclusion of cancers diagnosed within 12 months of entry.

### V. Conclusions

Animal and human ecological data suggest that dietary fat may play a major role in the aetiology of breast cancer. Human epidemiological evidence is, however, inconsistent and because of the homogeneity of dietary fat intake in most populations, does not exclude a strong association between fat consumption and disease risk. Evidence now exists indicating that the experimental study of the influence of dietary fat reduction on breast cancer incidence is feasible in a randomised controlled trial. Feasibility has been shown for the recruitment and retention of subjects, for dietary compliance and for the observed cancer risk of the subjects selected. Further, the sample size required for such a trial is attainable in Canada, and presumably elsewhere, by a multicentre trial. Such a trial would provide the strongest evidence available concerning the relationship of dietary fat intake to breast cancer risk, and is the only means to determine whether high risk subjects can reduce their risk by a modification in diet.

### References

BAILAR, J.C. & SMITH, E.M. (1986). Progress against cancer? *N. Engl. J. Med.*, **314**, 1226.

BOYD, N.F. & MCGUIRE, V. (1990). Evidence of lipid peroxidation in premenopausal women with mammographic dysplasia. *Cancer Lett.*, **50**, 31.

BOYD, N.F., COUSINS, M., BEATON, M., KRIUKOV, V., LOCKWOOD, G. & TRITCHLER, D. (1989*b*) Quantitative changes in dietary fat intake and serum cholesterol in women: results from a randomised controlled trial. *Am. J. Clin. Nutr.* (in press).

BOYD, N.F., COUSINS, M., BEATON, M. & 8 others (1988). Clinical trial of low fat, high carbohydrate diet in subjects with mammographic dysplasia: report of early outcomes. *J. Natl Cancer Inst.*, **80**, 1244.

BOYD, N.F., MCGUIRE, V., FISHELL, E., KRIUKOV, V., LOCKWOOD, G. & TRITCHLER, D. (1989*a*) Plasma lipids in premenopausal women with mammographic dysplasia. *Br. J. Cancer*, **59**, 766.

BOYD, N.F., O'SULLIVAN, B., CAMPBELL, J.E. & 4 others (1982). Mammographic signs as risk factors for breast cancer. *Br. J. Cancer*, **45**, 185.

BRISSON, J., MERLETTI, F., SADOWSKI, N.L. & 3 others (1982). Mammographic parenchymal patterns of the breast and breast cancer risk. *Am. J. Epidemiol.*, **115**, 428.

BRISSON, J., MORRISON, A.S. & KHALID, H. (1988). Mammographic parenchymal features and breast cancer in the breast cancer detection demonstration project. *J. Natl Cancer Inst.*, **80**, 1534.

BRISSON, J., VERREAULT, R., MORRISON, A.S., TENNINA, S. & MEYER, F. (1989). Diet, mammographic features of breast tissue, and breast cancer risk. *Am. J. Epidemiol.*, **130**, 14.

CARROLL, K.K., BRADEN, L.M., BELL, J.A. & 1 other (1986). Fat and cancer. *Cancer*, **58**, 1818.

COHEN, L.A. (1987). Diet and cancer. *Sci. Amer.*, **257**, 42.

DOLL, R. & PETO, R. (1981). *The causes of cancer: Quantitative estimates of avoidable risks of cancer in the United States.* Oxford University Press: Oxford.

GOODWIN, P.J. & BOYD, N.F. (1988). Mammographic parenchymal pattern and breast cancer risk: a critical appraisal of the evidence. *Am. J. Epidemiology*, **127**, 1097.

GOODWIN, P. & BOYD, N.F. (1987). A critical appraisal of the evidence that dietary fat intake is related to breast cancer risk in humans. *J. Natl Cancer Inst.*, **79**, 473.

HEGSTED, D.M., MCGAMBY, R.B., MYERS, M.L. & STARE, F.J. (1965). Quantitative effects of dietary fat on serum cholesterol in man. *Am. J. Clin. Nutr.*, **17**, 281.

HOLM, L.-E., CALLMER, E., HJALMAR, M.-L., LIDBRINK, E., NILSSON, B. & SKOOG, L. (1989). Dietary habits and prognostic factors in breast cancer. *J. Natl Cancer Inst.*, **81**, 1218.

HOWE, G.R., HIROMATA, T., HISLOP, T.G. & 9 others (1990). Dietary factors and risk of breast cancer: combined analysis of 12 case-control studies. *J. Natl Cancer Inst.*, **82**, 561.

KAGAWA, Y. (1978). Impact of Westernisation on the nutrition of Japanese: changes in the physique, cancer, longevity and centenarians. *Prev. Med.*, **7**, 205.

KEYS, A., ANDERSON, J.T. & GRANDE, F. (1965). Serum cholesterol response to changes in the diet. iv, particular saturated fatty acids in the diet. *Metabolism*, **14**, 776.

LEE-HAN, H., COUSINS, M., BEATON, M. & 4 others (1988). Compliance in a randomised clinical trial of dietary fat reduction in patients with breast dysplasia. *Am. J. Clin. Nutr.*, **48**, 575.

PRENTICE, R., KAKAR, F., HURSTING, S., SHEPPARD, L., KLEIN, R. & KUSHI, L.H. (1988). Aspects of the rationale for the Women's Health Trial. *J. Natl Cancer Inst.*, **80**, 802.

ROGERS, A.E. & LEE, S.Y. (1986). Chemically induced mammary gland tumors in rats: modulation by dietary fat. *J. Natl Cancer Inst.*, **80**, 255.

ROSE, D.P. Dietary factors and breast cancer. *Cancer Surveys*, **3**, 671.

SAFTLAS, A., WOLFE, J.N., HOOVER, R. & 4 others (1989). Mammographic patterns as indicators of breast cancer risk. *Am. J. Epidemiol.*, **129**, 518.

SAFTLAS, A.F. & SZKLO, M. (1987). Mammographic Parenchymal Patterns and Breast Cancer Risk. In: *Epidemiologic Reviews 1987*. Szklo, M., Gordis, L., Gregg, M.B. & Levine, M.M. (eds). Johns Hopkins University School of Hygiene and Public Health: Boston.

SCHATZKIN, A., GREENWALD, P., BYAR, D.P. & CLIFFORD, C. (1989). The dietary fat-breast cancer hypothesis is alive. *J. Am. Med. Assoc.*, **261**, 3284.

SELF, S., PRENTICE, R., IVERSON, D. & 10 others (1988). Statistical design of the Women's Health Trial, *Controlled Clinical Trials*, **9**, 119.

SILVERBERG, E. & LUBRA, J.A. (1988). Cancer statistics, 1988. *Ca- - A Cancer J. for Clin.*, **38**, 5.

TONIOLI, P., RIBOLI, E., PROTTA, F., CHARREL, M. & CAPPA, A.P.M. (1989). Calorie providing nutrients and risk of breast cancer. *J. Natl Cancer Inst.*, **81**, 278.

VERREAULT, R., BRISSON, J., DESCHENNES, I. & 3 others (1988). Dietary fat in relation to prognostic indicators in breast cancer. *J. Natl Cancer Inst.*, **80**, 819.

WELSCH, C.W., DEHOOG, J.V., O'CONNER, D.H. & SHEFFIELD, L.G. (1985). Influence of dietary fat levels on development and hormone responsiveness of the mouse mammary gland. *Cancer Res.*, **45**, 6147.

WELSCH, C.W. (1986). Interrelationships between dietary fat and endocrine processes in mammary gland tumorigenesis. In: *Dietary Fat and Cancer*. Ip, C., Birt, D.F., Rogers, A.E. & Mettlin, C. *Progress in Clinical and Biological Research*, **222**, 623.

WILLETT, W.C., STAMPFER, M.J., COLDITZ, G.A. & 3 others (1987). Dietary fat and the risk of breast cancer. *N. Engl J. Med.*, **316**, 22.

WILLETT, W.C. (1989). The search for the causes of breast and colon cancer. *Nature*, **338**, 389.

WOLFE, J.N., SAFTLAS, A.F. & SALANE, M. (1987). Mammographic parenchymal patterns and quantitative evaluation of mammographic densities: a case control study. *Am. J. Roentgenol.*, **148**, 1087.